# Follicle-stimulating hormone linked to cognitive decline and amyloid burden in postmenopausal women

**DOI:** 10.3389/fnagi.2025.1697255

**Published:** 2026-01-06

**Authors:** Sheng-Min Wang, Chaiho Jeong, Yoo Hyun Um, Dong Woo Kang, Sunghwan Kim, Soyoung Lee, Chang Uk Lee, Howard J. Aizenstein, Ki-Hyun Baek, Hyun Kook Lim

**Affiliations:** 1 Department of Psychiatry, Yeouido St. Mary’s Hospital, College of Medicine, The Catholic University of Korea, Seoul, Republic of Korea; 2 Division of Endocrinology and Metabolism, Department of Internal Medicine, Uijeongbu St. Mary's Hospital, College of Medicine, The Catholic University of Korea, Seoul, Republic of Korea; 3 Department of Psychiatry, St. Vincent Hospital, Suwon, Korea, College of Medicine, The Catholic University of Korea, Seoul, Republic of Korea; 4 Department of Psychiatry, Seoul St. Mary’s Hospital, College of Medicine, The Catholic University of Korea, Seoul, Republic of Korea; 5 Department of Psychiatry, Brigham and Women’s Hospital, Boston, MA, United States; 6 Department of Psychiatry, Harvard Medical School, Boston, MA, United States; 7 Department of Psychiatry, University of Pittsburgh, Pittsburgh, PA, United States; 8 Division of Endocrinology and Metabolism, Department of Internal Medicine, Yeouido St. Mary's Hospital, College of Medicine, The Catholic University of Korea, Seoul, Republic of Korea; 9 CMC Institute for Basic Medical Science, The Catholic Medical Center of The Catholic University of Korea, Seoul, Republic of Korea

**Keywords:** follicle-stimulating hormone, estrogen, cognitive impairment, amyloid retention, Alzheimer’s disease

## Abstract

**Introduction:**

Women have a higher risk of developing Alzheimer’s disease (AD) than men, with hormonal changes during menopause being a potential factor. However, the exact relationship between these hormonal changes, cognitive function, and AD pathology is not fully understood. This study investigates the differential associations between serum follicle-stimulating hormone (FSH) and estradiol levels with cognitive function and cerebral amyloid-βeta (Aβ) deposition, quantified using amyloid positron emission tomography, in postmenopausal women across the spectrum from cognitively normal aging to AD dementia.

**Methods:**

A total of 884 postmenopausal women, aged 60 years or older, were enrolled in the study. Participants were classified into three groups based on their cognitive function: cognitively normal (CN), mild cognitive impairment (MCI), and AD dementia.

**Results:**

Higher FSH levels were associated with poorer cognitive performance and greater cerebral Aβ deposition in postmenopausal women. FSH levels were highest in women with AD dementia, followed by those with MCI, and lowest in CN participants. No significant relationship was observed between estradiol levels and cognitive outcomes or Aβ burden. Further analysis showed a positive correlation between FSH levels and global as well as regional cerebral Aβ deposition. Mediation analysis indicated that FSH’s impact on cognitive function was mediated by cerebral Aβ burden. Estradiol levels, however, had no significant association with either cognitive performance or Aβ pathology.

**Discussion:**

Elevated FSH, not low E2, is linked to cognitive decline and Aβ pathology in postmenopausal women. FSH may be a key risk factor for cerebral Aβ deposition and cognitive decline in older women. Further research is needed to elucidate the mechanisms involved and explore hormonal interventions for AD.

## 1 Introduction

Alzheimer’s disease (AD) is a progressive neurodegenerative disorder characterized by cognitive dysfunction and impairment in daily living activities (Chin et al., 2024; Jack et al., 2024). Women face a significantly higher risk of developing AD, with estimates suggesting this risk to be two to three times greater than that for men (Snyder et al., 2016; Scheyer et al., 2018). Women account for approximately two-thirds of all AD cases, and projections suggest a near fourfold increase in AD cases by 2050, driven by population growth and aging, with women disproportionately affected (Yu et al., 2025). In addition, women often experience more severe AD pathology and faster cognitive decline compared to men, and postmenopausal age represents the period of steepest increase in AD incidence (Laws et al., 2016; Marongiu, 2019). These data underscore the clinical urgency of understanding endocrine drivers of AD risk in older women.

In humans, the menopausal transition is characterized by declining estradiol (E2) and rising follicle-stimulating hormone (FSH) levels, as defined in the STRAW+10 framework (Harlow et al., 2012). This hormonal shift has been suggested as a key factor contributing to the higher risk of AD observed in women compared with men. After menopause, ovarian estrogen synthesis falls sharply, and reduced serum E2 was long considered the main endocrine link between female sex and AD susceptibility (Scheyer et al., 2018; Nerattini et al., 2023). However, emerging evidence indicates that elevated FSH—not low E2—may act synergistically with inflammatory, lipid, and vascular pathways to promote cerebral Aβ deposition and neurodegeneration (Xue et al., 2025). These findings suggest that FSH is not merely a reproductive marker but an endocrine driver of AD-related pathology through extragonadal mechanisms and associated neuroendocrine and metabolic changes (Harlow et al., 2012).

Preclinical studies have shown that estrogen enhances memory and learning and can facilitate dendritic spine growth in the hippocampus and medial prefrontal cortex (Sheppard et al., 2019). Other studies suggest that estrogen has a neuroprotective effect by promoting brain-derived neurotrophic factors (Harte-Hargrove et al., 2013; Gao et al., 2022). However, more recent findings suggest that FSH, rather than estrogen or E2, is strongly associated with the onset of AD (Xiong et al., 2022; Xiong et al., 2023). An animal study illustrated that FSH acts directly on hippocampal and cortical neurons to accelerate amyloid-βeta (Aβ) and tau deposition, resulting in cognitive decline (Xiong et al., 2022). More importantly, suppressing FSH activity in these mice eliminated the AD-like characteristics by hindering the neuronal proinflammatory cytokines-activated transcription factor (C/EBPβ) and *δ*-secretase pathway. Another animal study further showed that the Apolipoprotein E (APOE) ε4 allele and FSH jointly trigger the activation of the C/EBPβ/δ-secretase pathway, facilitating the proteolytic fragmentation of Aβ precursor protein and tau, leading to the production of Aβ and the formation of neurofibrillary tangles (Xiong et al., 2023).

Despite these findings, the relationship between hormonal changes and AD pathology in women remains unclear. Studies investigating the association between AD incidence and post-menopausal estrogen decline in human samples have yielded conflicting results (Shumaker et al., 2004; O'Brien et al., 2014; Matyi et al., 2019). While one study demonstrated that estrogen replacement therapy correlated with better cognitive function in later life (Matyi et al., 2019), a meta-analysis found no association between postmenopausal estrogen therapy and reduced AD risk (O'Brien et al., 2014). Contrarily, some studies have suggested that estrogen therapy may even increase the incidence of mild cognitive impairment (MCI) and AD dementia in post-menopausal women (Shumaker et al., 2004). Further research suggests that the effects of hormone replacement therapy on AD-related brain changes in women may depend on several mediating factors, including the age at menopause, timing of hormone replacement initiation, duration of therapy, as well as variations in study design and population characteristics (Buckley et al., 2018; Sauve et al., 2024; Barth et al., 2025).

The role of FSH in the pathophysiology of AD is also obscure. During the perimenopause period, there is a phase where estrogen levels remain relatively stable even as serum FSH levels increase (Randolph et al., 2011), and women are known to experience transient cognitive decline during this perimenopause period (Epperson et al., 2013). Others postulated that FSH may contribute to AD pathology by targeting receptors in the hippocampus and cerebral cortex (Sauve et al., 2024). However, a more recent study showed no relations between FSH level and cognitive decline, although FSH level was associated with cerebral Aβ deposition in the frontal cortex (Nerattini et al., 2023).

A possible explanation for these contradictory results is that previous clinical studies did not utilize biomarker-based diagnosis to define AD. None of the estrogen studies used amyloid positron emission tomography (A-PET) or fluid biomarkers to diagnose their participants (Shumaker et al., 2004; Epperson et al., 2013; O'Brien et al., 2014). Regarding FSH studies, although one study used A-PET to measure cerebral Aβ deposition, it did not specify participants’ Aβ status as positive or negative. Moreover, the study was unable to categorize patients into different clinical stages because it did not measure global cognitive function using a comprehensive neuropsychological measure. Thus, previous studies were unable to elucidate whether the differences in serum FSH or estrogen in subjects with AD pathology correlated to cerebral Aβ burden, neurocognitive function, or a mixture of both.

To address this gap, we investigated whether serum FSH and estrogen levels differ according to the clinical stage of AD in older women. Additionally, we analyzed the associations of serum FSH and estrogen levels with cerebral Aβ deposition severity and neurocognitive function. We hypothesized that higher circulating FSH, but not estradiol, would be associated with greater cerebral amyloid burden and poorer cognitive performance in postmenopausal women.

## 2 Materials and methods

### 2.1 Subjects

Participants were drawn from the Catholic Aging Brain Imaging (CABI) database, an established institutional neuroimaging registry maintained at the Catholic Brain Health Center, Yeouido St. Mary’s Hospital, The Catholic University of Korea. The database includes clinical and imaging data obtained from patients who visited the outpatient memory clinic between 2017 and 2024 (Kim et al., 2024). The inclusion criteria for all subjects were: (1) biologically female (sex assigned at birth = female) (2) age ≥ 60 years, (3) no clinically significant psychiatric disorders (depressive disorder, schizophrenia, or bipolar disorder), and (4) completion of the Korean version of the Consortium to Establish a Registry for Alzheimer’s Disease (CERAD-K), which includes a verbal fluency test, the 15-item Boston Naming Test (BNT), the Korean version of the Mini-Mental State Examination (MMSE), word list memory, word list recall, word list recognition, constructional praxis, and constructional recall to diagnose their clinical staging (Lee et al., 2002). Additionally, all subjects were confirmed to be menopausal, defined as a minimum of 12 months since the last menstruation by self-report or had a clinical diagnosis of menopause documented at baseline.

The cognitively normal older adults (CN) showed normal cognitive functions in the CERAD-K. In terms of MCI, patients met the following criteria: (1) presence of memory complaints corroborated by an informant (a family member or caregiver who could reliably report on the patient’s cognitive and functional abilities); (2) objective cognitive impairment in more than one cognitive domain on CERAD-K (at least 1.0 standard deviation (SD) below age- and education-adjusted norms); (3) intact activities of daily living; (4) a Clinical Dementia Rating (CDR) of 0.5; and (5) not demented according to the Diagnostic and Statistical Manual of Mental Disorders (DSM)-V criteria (Saunders et al., 2018). Lastly, the patients with AD dementia met the probable AD criteria proposed by the National Institute of Neurological and Communicative Disorders and Stroke and the Alzheimer’s Disease and Related Disorders Association (NINCDS-ADRDA) (Dubois et al., 2007), as well as those proposed by the DSM-V with positive findings or high cerebral Aβ deposition in A-PET (Tay et al., 2015).

We excluded subjects with the followings: (1) systemic diseases that can cause cognitive impairment, such as thyroid dysfunction, severe anemia, and syphilis infection; (2) severe hearing or visual impairment; (3) history of head trauma and other neurological diseases that can cause cognitive impairment, such as brain tumor, encephalitis, and epilepsy; (4) clinically significant cerebral infarction or cerebral vascular disease; (5) prescription medications that may cause changes in cognitive function; (6) contraindications to MRI, including the presence of metallic implants, pacemakers, electronic devices incompatible with MRI, and severe claustrophobia; and (7) a history of or currently receiving hormone replacement therapy.

All procedures contributing to this work complied with the ethical standards of the relevant national and institutional committees on human experimentation and with the Helsinki Declaration of 1975, as revised in 2013. The study was reviewed and approved by the Institutional Review Board of The Catholic University of Korea (SC22RIDI0153). Informed consent, the process of obtaining voluntary agreement from participants after providing them with information about the study, was not required for our research. This waiver was granted by the institutional review board because the study was retrospective, utilizing previously collected medical records and imaging. Additionally, all data were anonymized prior to analysis to ensure participant confidentiality and privacy.

### 2.2 Amyloid-positron emission tomography and MRI image acquisition

All A-PET scans were conducted using ^18^F-flutemetamol (^18^F-FMM) under the standardized protocol (Sur et al., 2022; Kim et al., 2024). Image acquisition began 90 min after administering a bolus injection of 185 MBq (5.0 mCi) of ^18^F-FMM, cold mass <3 μg, with Biograph 40 TruePoint (Siemens Medical Solutions, Erlangen, Germany). PET data acquisition lasted 20 min and consisted of four 5-min frames. For attenuation correction and anatomical localization without contrast enhancement, low-dose CT scans were performed using settings of 120 kV and 70 mAs with a slice thickness of 3 mm. In terms of MRI, structural T1-weighted images were scanned using a 3 T Siemens MAGETOM Skyra machine and a 20-channel head and neck coil (Siemens Medical Solutions, Erlangen, Germany). The magnetization-prepared rapid gradient echo scan sequences were echo time = 2.6 ms, repetition time = 1940 ms, flip angle = 9°, field-of-view = 224 × 224 mm, matrix size of 256 × 256 with a slice thickness of 1.0 mm. All MRIs were acquired within 3 months prior to or following the A-PET scan.

### 2.3 Blinded visual analysis of A-PET

Two nuclear medicine physicians visually inspected and interpreted the A-PET images. They assessed cerebral Aβ retention by comparing the uptake intensity in the gray matter to that in the white matter across six specified brain regions: the frontal lobes, lateral temporal lobes, parietal lobes, anterior cingulate cortex, posterior cingulate cortex, and precuneus, and striatum (Collij et al., 2021). A scan was categorized as Aβ-positive if any of these regions showed uptake in the gray matter that was equal to or greater than the adjacent white matter. The visual interpretations were performed independently of the quantitative analysis and served as the reference standard for determining Aβ-positivity. Any discrepancies between the two raters were resolved through consensus.

### 2.4 Quantitative assessment of Aβ deposition

To quantify regional and global Aβ retention in the brain, we utilized SCALE PET (version 0.1.3.1, Neurophet Inc., Seoul, Republic of Korea), an automated segmentation software powered by a deep-learning model (Lee et al., 2022). The A-PET images were aligned to the T1-weighted MRI images through affine or non-linear co-registration to define regions of interest and correct for partial volume effects caused by cerebral atrophy. The software computed regional standardized uptake value ratios (SUVRs) for six cortical regions of interest (frontal lobes, lateral temporal lobes, parietal lobes, anterior cingulate cortex, posterior cingulate cortex, precuneus, and striatum) based on the Desikan–Killiany atlas, using the pons as the reference region (Thurfjell et al., 2014). The regional SUVRs were calculated as the mean uptake in each region of interest (e.g., frontal lobe) divided by the mean uptake in the pons. A global SUVR was calculated by averaging the SUVRs of the six cortical regions of interest. All SUVR values were derived from data acquired 90 min after the injection of ^18^F-FMM. Consistent with thresholds established in previous studies, a cutoff value of 0.62 for global SUVR was used to support the determination of amyloid positivity (Thurfjell et al., 2014). To ensure consistency and reliability, we excluded A-PET images with discordance between the visual readings.

### 2.5 FSH and E2 analysis and APOE genotyping

There are three major endogenous estrogens that have estrogenic hormonal activity: estrone (E1), E2, and estriol (E3) (Zhu et al., 2023). Among them, we selected E2 for measurement because it has garnered significant attention in the context of AD, being the predominant estrogen during reproductive years and dropping to prepubertal levels after menopause (Ali et al., 2023; Nerattini et al., 2023). Both E2 and FSH levels were determined from a single serum sample. Serum was collected from each woman between 10 a.m. and 3 p.m. Blood samples were centrifuged in a refrigerated centrifuge, and after separation, serum was frozen at −20 °C until assay. E2 and FSH were measured by radioimmunoassay using GammaPro (Kaien, Korea). Sensitivity or minimum detection limit for E2 and FSH was calculated at 4.0 pg./mL and 0.3 mIU/mL, respectively.

Plasma levels of E2 in postmenopausal women are typically very low, ranging from 2 to 21 pg./mL as determined by mass spectrometry-based methods. Consequently, a significant proportion of participants may exhibit E2 levels below the detection limit of the radioimmunoassay (Blair, 2010). For samples below the detection limit, commonly applied methods include replacing values with the lower limit of quantification, half the lower limit of quantification, or zero. In our primary analysis, we assigned a constant value of 3.90 pg./mL to participants whose E2 levels fell below the detection limit of 4.0 pg./mL, as this value is closer to the lower limit of quantification. To ensure the robustness of our findings, we also conducted an additional sensitivity analysis using a value of 2.0 pg./mL, which corresponds to half the lower limit of quantification.

For APOE genotyping, venous blood samples were collected in EDTA tubes at baseline. Genomic DNA was extracted from peripheral whole blood using standard procedures. APOE genotypes (ε2, ε3, ε4) were determined based on the rs429358 and rs7412 polymorphisms using a commercially available allelic discrimination assay. Participants were classified as APOE ε4 carriers (≥1 ε4 allele) or non-carriers (no ε4 allele), and this binary APOE ε4 status was used in all analyses.

### 2.6 Statistical analysis

We used the free and open-source data analysis tool Jamovi (Version 2.3.18.0), along with the Python packages SciPy (version 1.10.0) and Pingouin (version 0.5.3), to conduct statistical analyses. Analysis of variance (ANOVA) or analysis of covariance (ANCOVA) was employed to assess potential differences among the three groups (CN, MCI, and AD) for continuous variables, while the Chi-square test was used for categorical variables. When group differences were statistically significant, Bonferroni tests were conducted for *post-hoc* analysis. Pearson correlation was utilized to examine bivariate associations, and partial correlations were used to adjust for covariates (i.e., age) when appropriate. A two-tailed *α* level of 0.05 was set to indicate statistical significance for all statistical tests.

To investigate factors associated with cerebral Aβ positivity, we first performed univariate logistic regression analyses, with Aβ status (positive vs. negative) as the dependent variable and each predictor (age, education, APOE ε4 carrier status, FSH, and E2) entered separately. Predictors that reached statistical significance in the univariate analyses were then entered into a multivariate logistic regression model to identify independent factors associated with Aβ positivity. Similarly, predictors of global mean SUVR were examined using a two-step approach. We first conducted univariate linear regression analyses for each predictor. Variables significant in univariate testing were subsequently included in a multivariate linear regression model to assess independent associations with global mean SUVR. Model assumptions (linearity, normality, and homoscedasticity of residuals, and absence of multicollinearity) were evaluated using residual plots, Q–Q plots, and variance inflation factors, and no major violations were detected.

A mediation analysis was conducted to examine whether the association between FSH levels and cognitive performance (CERAD-K total score) was mediated by cerebral Aβ burden (global mean SUVR). This analysis was performed using the Mediation module in Jamovi, which estimates ordinary least-squares linear regression models for each path. All paths (FSH → global mean SUVR, global mean SUVR → CERAD-K, and FSH → CERAD-K) were adjusted for age and APOE ε4 carrier status. The indirect (mediated) effects were estimated using a bootstrapping procedure with 5,000 resamples, and statistical significance was considered when the 95% confidence interval (CI) did not include zero.

## 3 Results

### 3.1 Baseline demographic and clinical data

A total of 884 participants were analyzed (Table 1). All variables were normally distributed. Age and education differed significantly among groups, with the CN group being younger and more educated than the MCI and AD dementia groups (*p* < 0.05, ANOVA, Bonferroni correction), but MCI and AD dementia did not differ. APOE ε4 frequency was highest in AD dementia and lowest in CN. CERAD-K scores were highest in CN, followed by MCI, and lowest in AD dementia (*p* < 0.001, ANOVA; *p* < 0.05, *post-hoc*).

**Table 1:** Demographic and clinical characteristics of total participants.

Variable	Total (*N* = 884)		CN (*N* = 273)		MCI (*N* = 477)		AD dementia (*N* = 134)		*p*-value
	Mean	SD	Mean	SD	Mean	SD	Mean	SD	
Age (years ± SD)	76.94	7.17	73.3	6.80	78.6	6.17	78.77	8.40	<0.001
Education (years ± SD)	8.92	5.54	9.82	4.49	8.40	5.08	8.84	8.13	<0.001
APOE ε4 (Yes, %)^+^	312, 35.29%		67, 24.5%%		177, 37.11%		68, 50.74%		<0.001
CERAD-K Battery (SD)									
Verbal fluency	10.22	4.89	14.48	4.12	9.28	3.52	4.76	3.16	<0.001
BNT	9.68	3.35	12.14	1.95	9.25	2.88	6.05	3.31	<0.001
MMSE	22.30	6.04	27.91	1.13	21.68	4.07	13.15	5.68	<0.001
Word list memory	13,851	5.33	18.93	3.51	12.82	3.58	7.19	4.02	<0.001
Constructional praxis	8.80	2.01	10.04	1.12	8.81	1.75	6.89	2.57	<0.001
Word list recall	3.35	2.57	6.19	1.70	3.02	1.89	0.68	0.92	<0.001
Word list recognition	6.35	3.13	9.13	0.97	6.05	2.51	2.01	2.25	<0.001
Constructional recall	3.25	3.23	6.45	2.62	2.57	2.41	1.16	1.76	<0.001
CERAD-K total score	52.15	17.91	70.30	11.23	50.19	14.08	27.17	11.70	<0.001

^+^Statistical analysis performed with the Chi-squared test. AD, Alzheimer’s disease; BNT, 15-Item Boston Naming Test; CERAD-K, The Korean Version of Consortium to Establish A Registry for Alzheimer’s Disease; CN, Cognitively normal older adults; MCI, Mild cognitive impairment; MMSE, Mini Mental Status Examination; SD, standard deviation; Verbal fluency.

### 3.2 FSH, E2, and clinical stage of AD

**Figure 1:**
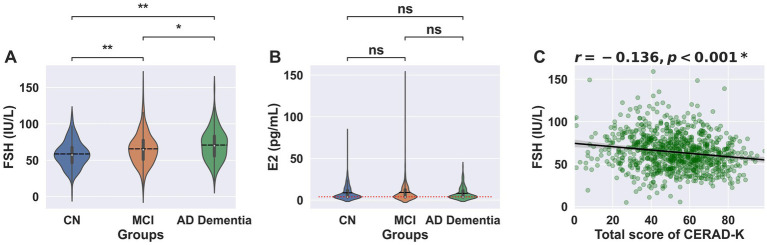
FSH and E2 levels according to the clinical stage of AD. Violin plots showing **(A)** FSH levels and **(B)** E2 levels across CN, MCI, and AD dementia groups. Vertical lines represent the interquartile range, medians are marked by a white dot, and means are indicated by a black dashed horizontal line. **(A)** ^*^p < 0.05 and ^**^p < 0.01 in *post-hoc* analysis using Bonferroni correction for analysis of variance (ANCOVA), **(B)** Red dotted horizontal line represents the lower detection limit of E2. **(C)** ^*^Partial correlation analysis with age as the covariate (*N* = 884). AD, Alzheimer’s disease; CERAD-K, The Korean Version of Consortium to Establish A Registry For Alzheimer’s Disease; CN, cognitive normal older adults; FSH, Follicle-stimulating hormone; MCI, Mild cognitive impairment; ns, statistically not significant.

There was a group difference in serum FSH level (*p* < 0.001 for ANOVA). Since the baseline demographic data showed a significant group difference in age, we conducted ANCOVA with age as a covariate, in addition to ANOVA. The results of ANCOVA showed that the group difference in FSH remained significant even after adjusting for age (*p* < 0.001 for ANCOVA). *Post-hoc* analysis showed that FSH level was highest in AD dementia (mean ± standard deviation = 70.9 ± 21.10), followed by MCI (65.62 ± 21.93), and was lowest in CN (58.55 ± 17.72) (Figure 1A). In contrast, there was no group difference in serum E2 level (CN vs. MCI vs. AD dementia; 8.69 ± 8.36 vs. 9.17 ± 11.29 vs. 8.14 ± 7.23, *p* = 0.586 for ANOVA) (Figure 1B), and the group difference remained statistically insignificant even after adjusting for age (*p* = 0.638 for ANCOVA).

### 3.3 Association between FSH or E2 with neuropsychological measures

Initial correlation analysis showed a significant positive correlation between age and FSH (*r* = 0.104, *p* < 0.01). Thus, we conducted partial correlation analysis with age as a covariate. FSH showed a negative association with the CERAD-K total score (*r* = −0.136, *p* < 0.001, Figure 1C) and its sub-measures (Supplementary Figures 1A–G), including executive function (*r* = −0.112, *p* < 0.001), language function (*r* = −0.101, *p* = 0.002), MMSE (*r* = −0.148, *p* < 0.001), visuospatial function (*r* = −0.070, *p* = 0.05), and memory (*r* = −0.197, *p* < 0.001). However, E2 showed no significant association with the CERAD-K total score (*r* = −0.007, *p* = 0.828; Supplementary Figure 2A) or with any of the neuropsychological subdomains, including executive function (*r* = 0.009, *p* = 0.775), language (*r* = −0.025, *p* = 0.452), MMSE (*r* = 0.018, *p* = 0.594), visuospatial ability (*r* = 0.003, *p* = 0.927), and memory (*r* = 0.031, *p* = 0.353).

### 3.4 Association between FSH or E2 with cerebral Aβ deposition

**Figure 2:**
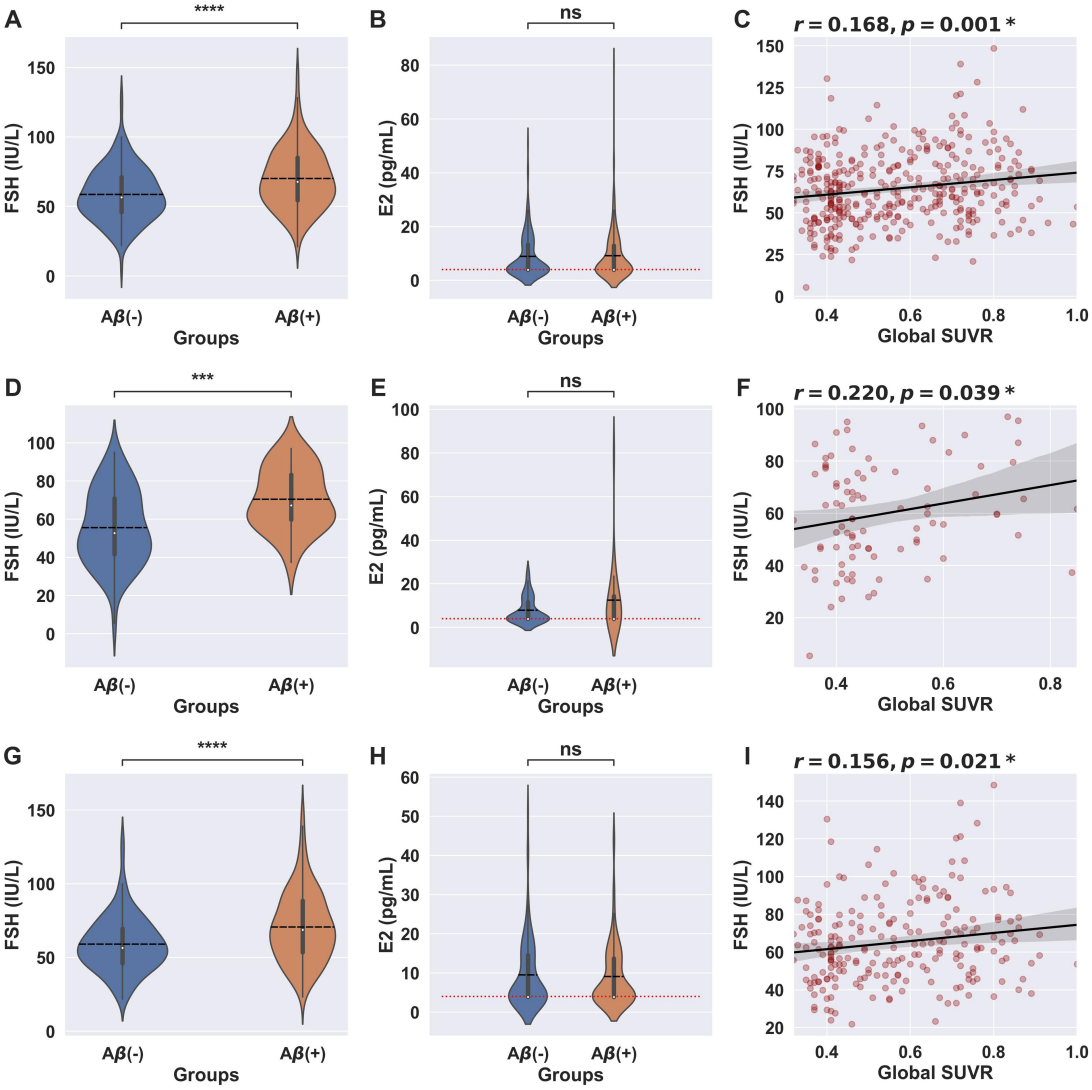
FSH and E2 levels according to the cerebral Aβ deposition. Violin plots showing FSH level in Aβ- and Aβ+ group for **(A)** all clinical stages (*N* = 367), **(D)** CN (*N* = 88), and **(G)** MCI (*N* = 221). Vertical lines represent the interquartile range, medians are marked by a white dot, and means are indicated by a black dashed horizontal line. ^***^p < 0.001, ^****^p < 0.0001 for Student’s *T*-test. Violin plots showing E2 level in Aβ- and Aβ+ group for **(B)** all clinical stages (*N* = 367), **(E)** CN (*N* = 88), and **(H)** MCI (*N* = 221). The red dotted horizontal line represents the lower detection limit of E2. Correlation analysis for **(C)** all clinical stages (*N* = 367), **(F)** CN (*N* = 88), and **(I)** MCI (*N* = 221). *Indicates statistically significant in Pearson correlation analysis. ns, statically not significant.

Among 884 participants, 367 underwent A-PET studies (Table 2). The Aβ- and Aβ+ groups did not differ in age or education. However, neuropsychological measures were statistically lower in the Aβ + group compared to the Aβ-group. The APOE ε4 allele was more frequent in the Aβ + group (54.8%) than in the Aβ-group (22.54%). The Aβ-group had a lower FSH level than the Aβ + group (58.60 ± 19.11 vs. 70.12 ± 21.75, *p* < 0.0001), but the two groups did not differ in E2 levels (8.90 ± 7.96 vs. 9.17 ± 9.26, *p* = 0.873) (Figures 2A,B). Additionally, global SUVR (*r* = 0.168, *p* = 0.001) (Figure 2C) and regional SUVR of the frontal lobe (*r* = 0.159, *p* = 0.002), parietal lobe (*r* = 0.169, *p* = 0.001), posterior cingulate cortex/precuneus (*r* = 0.167, *p =* 0.001), lateral temporal lobe (*r* = 0.172, *p* < 0.001), and anterior cingulate cortex (*r* = 0.153, *p* = 0.003) showed a positive correlation with FSH levels (Supplementary Figures 3A–E). However, E2 levels did not have significant association with global SUVR (*r* = 0.006, *p* = 0.903; Supplementary Figure 2B) and regional SUVRs (frontal lobe: *r* = 0.002, *p* = 0.976; parietal lobe: *r* = 0.009, *p* = 0.938; posterior cingulate cortex/precuneus: *r* = 0.004, *p* = 0.938; lateral temporal lobe: *r* = 0.002, *p* = 0.938; anterior cingulate cortex: *r* = 0.015, *p =* 0.779).

**Table 2:** Demographic and clinical characteristics of participants having A-PET results.

Variable	Aβ negative (*N* = 179)		Aβ positive (*N* = 188)		*p* value
	Mean	SD	Mean	SD	
Age (years ± SD)	76.16	7.05	76.88	6.73	0.323
Education (years ± SD)	9.29	4.92	9.94	7.43	0.334
APOE ε4 (Yes, %)	39, 22.54%		103, 54.8%		<0.001*
SUVR (average)	0.423	0.06	0.700	0.09	<0.001
CERAD-K battery (SD)					
Verbal fluency	11.22	4.74	9.63	4.77	<0.01
BNT	10.48	2.99	9.35	3.46	<0.001
MMSE	23.83	4.94	21.04	5.87	<0.01
Word list memory	15.00	5.15	12.53	4.86	<0.001
Constructional praxis	9.32	1.70	8.82	1.94	<0.05
Word list recall	4.02	2.78	2.49	2.22	<0.001
Word list recognition	6.82	2.94	5.28	3.16	<0.001
Constructional recall	4.25	3.41	2.12	2.65	<0.001
CERAD-K total score	56.53	16.59	48.01	16.63	<0.001

*Statistical analysis performed with Chi-square test. Aβ negative, low cerebral Aβ deposition in amyloid-Positron Emission Tomography Using 18F-Flutemetamol; Aβ positive, high cerebral Aβ deposition in amyloid-Positron Emission Tomography Using 18F-Flutemetamol; BNT, 15-Item Boston Naming Test; CERAD-K, The Korean Version of Consortium to Establish A Registry for Alzheimer’s Disease; CN, cognitively normal older adults; MCI, mild cognitive impairment; MMSE, Mini Mental Status Examination; NS, not significant, SD, standard deviation; SUVR, standardized uptake value ratio.

We conducted a subgroup analysis in subjects with CN and MCI. In subjects with CN, the Aβ-group (*N* = 63) had lower FSH level than the Aβ + group (*N* = 25) (55.54 ± 19.4 vs. 70.5 ± 15.9, *p* < 0.001), but the two groups did not differ in E2 level (8.04 ± 6.96 vs. 12.5 ± 16.05, *p =* 0.061) (Figures 2D,E). In addition, the global SUVR (Figure 2F; *r* = 0.220, *p* = 0.039) showed a positive correlation with the FSH levels. However, E2 levels did not have a significant association with cerebral Aβ deposition levels (global SUVR: *r* = 0.127, *p* = 0.239). The same trends of results were found in subjects with MCI. The Aβ- (*N* = 105) group had a lower FSH level than the Aβ + group (*N* = 116) (58.97 ± 18.77 vs. 70.71 ± 23.81, *p* < 0.001), but the two groups did not differ in E2 levels (9.53 ± 8.81 vs. 9.10 ± 7.94, *p =* 0.703) (Figures 2G,H). Lastly, the global SUVR (*r* = 0.156, *p* = 0.021) (Figure 2I) showed a positive association with FSH levels but with E2 levels (*r* = −0.036, *p* = 0.599).

### 3.5 Association among FSH, age, cerebral Aβ deposition, and neuropsychological measures

**Figure 3:**
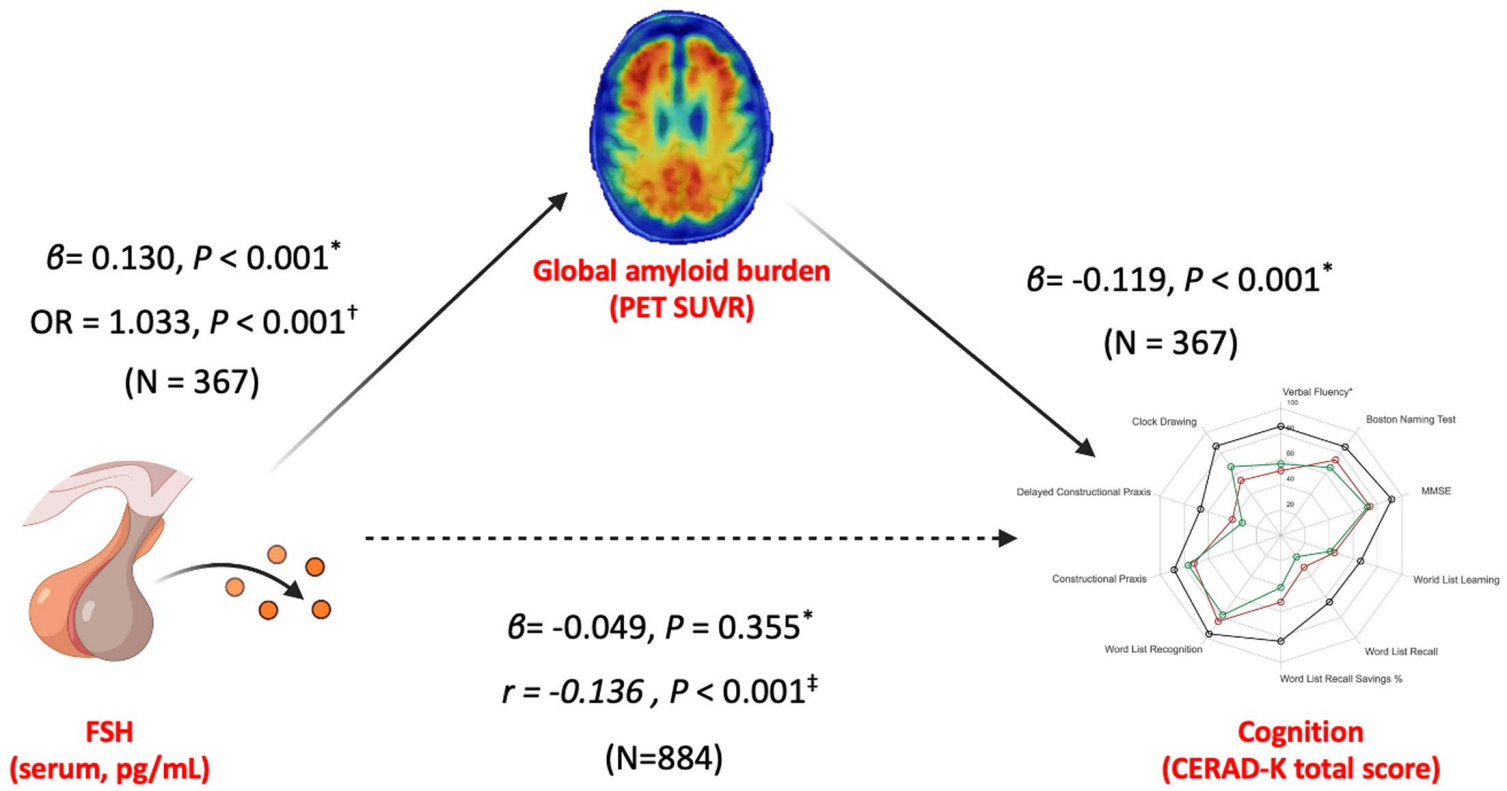
Integrated associations and mediation pathway among serum FSH, cerebral amyloid burden, and cognitive performance in postmenopausal women. The top image represents cerebral Aβ retention in global mean SUVR values from all subjects with A-PET results (*N* = 367), the left image represents FSH levels, and the right image represents cognitive function in CERAD-K total scores. ^*^Indicates mediation analysis. ^†^Indicates multivariate regression.

FSH levels were significantly associated with cerebral Aβ deposition and neuropsychological measures, prompting a mediation analysis to explore the process by which one variable affects another. Figure 3 shows the mediation analysis results, with FSH level as the independent variable and CERAD-K total scores as the dependent variable. The proposed mediator was the global cerebral Aβ retention (global mean SUVR value), given that cerebral Aβ retention is known to be an important risk factor for cognitive decline (Wirth et al., 2013). The analysis revealed no significant direct effect of FSH levels on CERAD-K total scores (*β* = −0.924, *p* = 0.355). However, the effect of FSH levels on CERAD-K total scores was mediated by global mean SUVR values (*β* = −0.0432, *p* = 0.006).

We also conducted a logistic regression analysis to investigate whether higher FSH levels were associated with increased odds of cerebral Aβ positivity. Among age, APOE ε4, FSH, and E2, univariate analysis identified APOE ε4 and FSH, but not E2 or age, as potential factors associated with cerebral Aβ positivity (Table 3). Multivariate analysis confirmed that FSH levels and APOE ε4 had significant impacts on cerebral Aβ positivity [odds ratio (OR) = 1.033, 95% CI: 1.020–1.046, *p* < 0.001].

**Table 3:** Analysis of factors associated with cerebral Aβ positivity.

Univariate regression analysis of factors associated with cerebral Aβ positivity				
Variable	Odd ratio (95% CI)	*p*-value	Pseudo-*R*^2^	AUC
APOE ε4				
No	1 (ref.)			
Yes	4.37 (1.014, 1.935)	*p* < 0.001	0.151	0.667
Age	1.015 (−0.0147, 0.045)	*p* = 0.32	0.0036	0.527
FSH	1.028 (0.017, 0.0391)	*p* < 0.001	0.0989	0.653
E2	1.00 (−0.0202, 0.0274)	*p* = 0.767	0.0003914	0.498

Multivariate regression analysis of factors associated with cerebral Aβ positivity				
APOE ε4				
No	1 (ref.)			
Yes	5.16 (1.49, 2.133)	*p* < 0.001	0.252	0.751
FSH	1.033 (0.02, 0.045)	*p* < 0.001		

AUC, area under the ROC curve.

To examine potential moderating effects, interaction terms were tested for FSH × APOE ε4 and FSH × age; neither interaction reached statistical significance (*p* = 0.54 and *p* = 0.814, respectively), indicating that the association between FSH and Aβ positivity was not significantly modified by APOE genotype or age. Potential confounders, including age, education, and APOE ε4, were also tested; age and education were not significant predictors, and their inclusion did not materially change the results. In stratified analyses by APOE ε4 carrier status, higher FSH levels were significantly associated with cerebral Aβ positivity in both non-carriers (Estimate = 0.029, *p* < 0.001; *R*^2^McF = 0.083) and carriers (Estimate = 0.040, *p* < 0.001; *R*^2^McF = 0.100), after adjusting for age, confirming that the FSH–Aβ association was consistent across APOE ε4 genotypes.

Finally, a linear regression analysis with global mean SUVR values as the dependent variable was conducted. Consistent with the logistic regression analysis, the results showed that APOE ε4 (*β* = 0.653, *p* < 0.001) and FSH (*β* = 0.169, *p* < 0.001) were significant predictors of global mean SUVR values, and the overall model explained approximately 12.4% of the variance in Aβ burden (adjusted *R*^2^ = 0.124). However, neither E2 nor age was a significant factor associated with global mean SUVR values.

### 3.6 Sensitivity analysis using half the lower limit of quantification for below the detection limit E2 values

For E2 values below the detection limit, we conducted sensitivity analysis after replacing them with half the lower limit of quantification, which is 2.0 pg./mL. The results were consistent with the main findings, which used 3.90 pg./mL as the replacement. The results showed no significant differences in E2 levels among clinical stages of AD (CN vs. MCI vs. AD dementia; 7.54 ± 9.05 vs. 7.98 ± 11.86 vs. 6.91 ± 7.98, *p* = 0.362 for ANOVA). In addition, there were no differences between the Aβ-negative (*N* = 179) and Aβ-positive (*N* = 188) in E2 levels (7.79 ± 8.68 vs. 8.04 ± 9.91, *p* = 0.975).

### 3.7 Sensitivity analysis excluding participants with E2 levels below the detection limit

**Figure 4:**
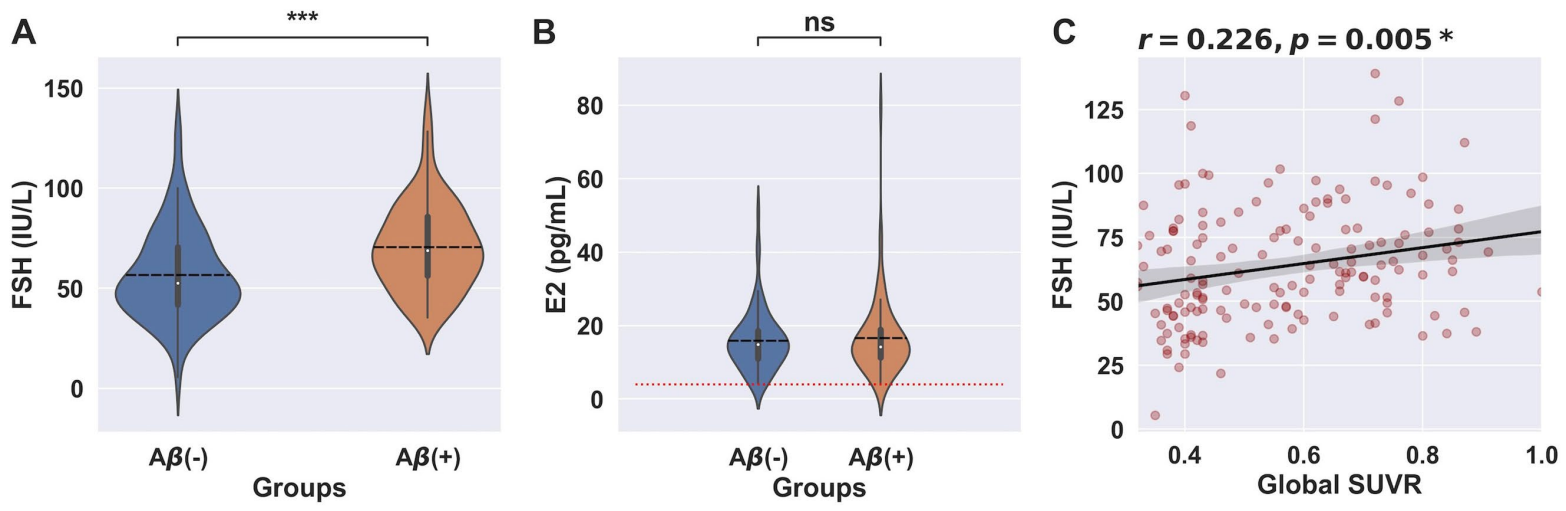
FSH and E2 levels according to cerebral Aβ deposition in an analysis excluding participants with E2 levels below the detection limit. Violin plots showing (**A**) FSH levels and (**B**) E2 levels in Aβ- (*N* = 75) and Aβ+ (*N* = 78) groups for all clinical stages. Vertical lines represent the interquartile range, medians are marked by a white dot, and means are indicated by a black dashed horizontal line. (**A**) ^*^^*^^*^p < 0.001 for Student’s T-test, (**B**) Red dotted horizontal line represents the lower detection limit of E2. (**C**) ^*^Statistical analysis done using Pearson correlation.

In our results, 62.2% of subjects had E2 levels below the detection limit of 4 pg./mL. To address this, a sensitivity analysis was conducted, excluding these participants. Consistent with the main results, the analysis showed no significant differences in E2 levels across the clinical stages of AD (*p* = 0.362), while FSH levels remained significantly different (*p* < 0.001). Similarly, FSH levels were significantly lower in the Aβ-group compared to the Aβ+ group (*p* < 0.001), with no significant differences observed in E2 levels (*p* = 0.873). FSH level also showed positive correlation with global SUVR (*r* = 0.226, *p* = 0.005) (Figures 4A–C).

## 4 Discussion

The role of female sex in increased AD incidence remains a focus of research, but the mechanisms underlying female-specific AD pathology are not fully understood. To our knowledge, this is the first study to investigate the associations among FSH, E2, clinical stages of AD, and the severity of cerebral Aβ deposition in postmenopausal women across the spectrum from cognitively normal aging to AD dementia. Our results revealed that in women, AD pathology was associated with FSH levels but not with E2 levels.

We report novel findings that the FSH levels were highest in the AD dementia group, followed by MCI, and lowest in CN. Higher FSH correlated negatively with neuropsychological scores, whereas E2 showed neither significant group differences according to the clinical stage of AD nor significant associations with cognitive functions. Consistent with our findings, an animal study demonstrated that lowering FSH improved cognition and reduced AD pathology (Xiong et al., 2022). However, the mechanisms linking high FSH to cognitive decline remain unclear.

Emerging evidence suggests several biological pathways through which elevated FSH may contribute to Alzheimer’s pathology. FSH receptors have been identified in hippocampal and cortical neurons, where activation can promote β-amyloid generation and neuroinflammatory responses. In postmenopausal women, rising FSH coincides with declining estrogen, leading to altered glucose metabolism and mitochondrial energy deficits in the brain, as shown by reduced glucose uptake and increased white-matter lipid utilization during the menopausal transition (Moutinho, 2025). These bioenergetic changes can exacerbate oxidative stress and amyloidogenic processes. Moreover, FSH may indirectly impair cerebrovascular and endothelial function, further compromising neuronal homeostasis (Ferretti et al., 2018). Interactions between FSH signaling, residual estrogens, and APOE genotype could amplify these effects, suggesting a multifactorial link between gonadotropin dysregulation, metabolic vulnerability, and female-specific AD risk.

A previous study reported an FSH-Aβ association in the frontal lobe only (Nerattini et al., 2023). We advanced previous research by showing that the FSH levels had a positive association with the global cerebral Aβ burden as well as the regional cerebral Aβ deposition of all regions, except for the striatum. The previous study did not specify Aβ deposition as either high or low, so it was not able to correctly elucidate whether the FSH level differed according to the cerebral Aβ deposition status (Nerattini et al., 2023). Thus, we are the first to report that FSH levels are higher in women with greater cerebral Aβ deposition than in those with lower Aβ deposition. Our findings further expand previous research by demonstrating that FSH levels are highest in postmenopausal women with AD and lowest in those with CN. Moreover, FSH levels remained positively associated with Aβ deposition within the same clinical stage. Taken together, our results suggest that FSH is linked not only to the severity of cerebral Aβ deposition but also to the clinical stages of AD.

One notable finding was the lack of significant associations between E2 levels and either neuropsychological measures or cerebral Aβ deposition. The relationship between E2 and AD pathologies has shown conflicting results. While some studies report that lower E2 levels in postmenopausal women are linked to worse cognitive function, (Xu et al., 2024) others find no such association (Mihalj et al., 2014). The reasons for these discrepancies are unclear, but recent research suggests multiple factors may influence the estrogen-AD relationship (Valencia-Olvera et al., 2023). For instance, estrogen may have a biphasic effect on AD pathology—neuroprotective in healthy neurons but neurotoxic in compromised cells (Brinton, 2005). Additionally, women who experience menopause at a later age tend to have elevated estrogen levels during the period when dementia-related changes occur, potentially leading to neurotoxic rather than neuroprotective effects in older age. Our participants may have had a higher average menopause age than those in previous studies, but we could not assess this due to the cross-sectional study design. Regardless, our findings contribute to the growing understanding of hormonal influences on AD and highlight the potential role of FSH, rather than E2, in linking cognitive decline and Aβ pathology in postmenopausal women. However, further research is needed to elucidate the underlying mechanisms and explore potential hormone-targeted therapies for AD.

While our study focused on E2, other estrogens, such as E1, may also influence AD pathogenesis. As the predominant estrogen in postmenopausal women, E1 is derived from androstenedione and has weaker estrogenic activity than E2 (Motlani et al., 2023). Its role in neuroprotection or neurodegeneration remains unclear, but the E1-to-E2 balance may offer insights into hormonal regulation in AD. Future studies should explore whether E1, alone or with E2, contributes to cognitive decline or Aβ pathology, potentially revealing new therapeutic targets.

Another factor warranting investigation is vascular dysfunction in the relationship between FSH, cognition, and Aβ deposition. FSH may impair vascular integrity by promoting endothelial dysfunction and oxidative stress (Wang et al., 2017; Ali et al., 2023), both of which are linked to AD progression (Polidori and Pientka, 2012). Elevated FSH levels in postmenopausal women could thus contribute to Aβ deposition and cognitive decline by disrupting vascular homeostasis. However, further research is needed to clarify FSH’s impact on vascular health and its role in AD.

A study using ovariectomized mice as a postmenopausal model demonstrated that FSH directly targets hippocampal and cortical neurons, accelerating Aβ deposition and impairing cognition (Xiong et al., 2023). However, no previous studies were able to illustrate this hypothetical model in humans. By conducting a mediation analysis, we found that the association between FSH and cognitive performance was statistically mediated by global mean SUVR values and cerebral Aβ deposition. In addition, FSH itself was not directly associated with cognition after accounting for cerebral Aβ burden. Since pathophysiological changes associated with AD begin decades before the onset of cognitive decline (Sperling et al., 2011), our findings might suggest that FSH elevation may contribute to the early pathological processes of AD.

Logistic regression analysis showed that FSH levels (OR = 1.033) and APOE ε4 (OR = 5.16), but not age or E2 levels, had significant impacts on cerebral Aβ positivity. In line with logistic regression analysis, our linear regression also showed that APOE ε4 (*β* = 0.657) and FSH (*β* = 0.169, *p* < 0.001), but not E2 or age, were associated with global mean SUVR values. Although FSH’s effect size is smaller than that of APOE ε4, our findings are the first to show that elevated FSH in postmenopausal women may be a risk factor for cerebral Aβ deposition. From another perspective, Aβ deposition is considered the earliest pathological hallmark of AD and an upstream event leading to tau accumulation and cognitive decline (Sanchez-Soblechero et al., 2024). Thus, FSH blockade might be an important target for slowing or preventing the onset of AD (Xiong et al., 2022). However, longitudinal and interventional studies are needed to confirm this hypothesis.

Compared with prior research, this study offers several methodological and conceptual advances. First, it includes a substantially larger cohort (*N* = 884) representing diverse cognitive stages, from cognitively normal older adults to those with AD dementia, providing stronger statistical power and generalizability than earlier hormonal or imaging studies. Second, cerebral Aβ deposition was quantified using amyloid PET, allowing biomarker-based classification rather than clinical diagnosis alone. Third, by simultaneously evaluating both FSH and estradiol, the study distinguishes their independent associations with cognition and amyloid pathology in postmenopausal women. Fourth, mediation and stratified analyses incorporating APOE ε4 and age revealed that the association between FSH and cognition operates indirectly through Aβ burden and is consistent across genotypes. Together, these strengths provide novel human evidence linking gonadotropin dysregulation to amyloid pathology, offering a mechanistic framework that extends prior preclinical and small-scale human studies.

This study has several limitations. First, its cross-sectional design limits causal inferences, as it only establishes correlations. Longitudinal studies are needed to clarify how hormonal changes contribute to cognitive decline and cerebral Aβ deposition in AD. Second, while radioimmunoassay is a widely used method, its detection limit (4 pg./mL) restricts the precise quantification of very low E2 levels in postmenopausal women. To address this, values below the detection limit were replaced with a constant value, a common practice that may introduce measurement bias by masking true variability. However, the proportion of participants with undetectable E2 levels was comparable across clinical groups and A-PET status, and sensitivity analyses excluding these participants yielded consistent results. Future studies using more sensitive methods, such as mass spectrometry, are needed to confirm these findings. Third, we lacked data on age at menopause, a key factor influencing hormonal levels and AD pathology. Future research should incorporate detailed hormonal histories to refine these associations. Fourth, SHBG, which regulates the bioavailability of estradiol, was not measured in this study. Future research incorporating SHBG levels would allow more precise evaluation of estrogen-related effects. Fifth, the registry did not systematically capture vascular risk factors such as hypertension, diabetes, dyslipidemia, or smoking. These factors are well-established contributors to cognitive decline and Aβ pathology, and their absence limits our ability to fully adjust for potential confounding. Residual confounding by unmeasured vascular or lifestyle variables therefore cannot be excluded. Sixth, we could not examine the mechanisms underlying gender differences in hormonal influences on AD pathology. Translational studies are needed to explore how gender, Aβ pathology, and cognitive decline interact in both sexes. Finally, the absence of tau-PET and plasma tau measurements prevented us from assessing FSH and E2 associations with tau pathology. Future studies should integrate tau biomarkers to provide a more comprehensive understanding of hormonal influences on AD progression.

In conclusion, our findings suggest that high FSH levels, rather than low E2 levels, are associated with increased cerebral Aβ burden and poorer cognitive performance in older women. We found that FSH levels correlated positively with both cognitive function and cerebral Aβ deposition, and that their impact on cognition was mediated by increased global cerebral Aβ burden. Additionally, elevated FSH levels were linked to higher odds of cerebral Aβ positivity. Taken together, these findings suggest that FSH may be a key risk factor for cerebral Aβ deposition and cognitive decline in older women.

## Data Availability

The raw data supporting the conclusions of this article will be made available by the authors, without undue reservation.
