# Supplementary material for: Follicle-stimulating hormone linked to cognitive decline and amyloid burden in postmenopausal women
Source: Front Aging Neurosci. 2026 Jan 6;17:1697255. doi: 10.3389/fnagi.2025.1697255 (PMC12816383; doi:10.3389/fnagi.2025.1697255)
Supplement: Supplementary file 1 [file Data_Sheet_1.docx]

**Supplementary Materials for “Follicle-Stimulating Hormone Linked to Cognitive Decline and Amyloid Burden in Postmenopausal Women” by Wang et al.**

- **Supplementary Figures**

1. Figure S1. Association between FSH and cognitive function

2. Figure S2. Association between E2 with cognitive function and cerebral Aβ deposition

3. Figure S3. Association between FSH with cerebral Aβ deposition

**Supplementary Figures**

**Figure S1. Association between FSH and cognitive function**

The results of partial correlation analysis showed that the FSH level had a negative association with the sub-measures of CERAD-K including A) executive function subscore; B) language function subscore; C) MMSE subscore; D) visuospatial function subscore, and E) memory subscore

**
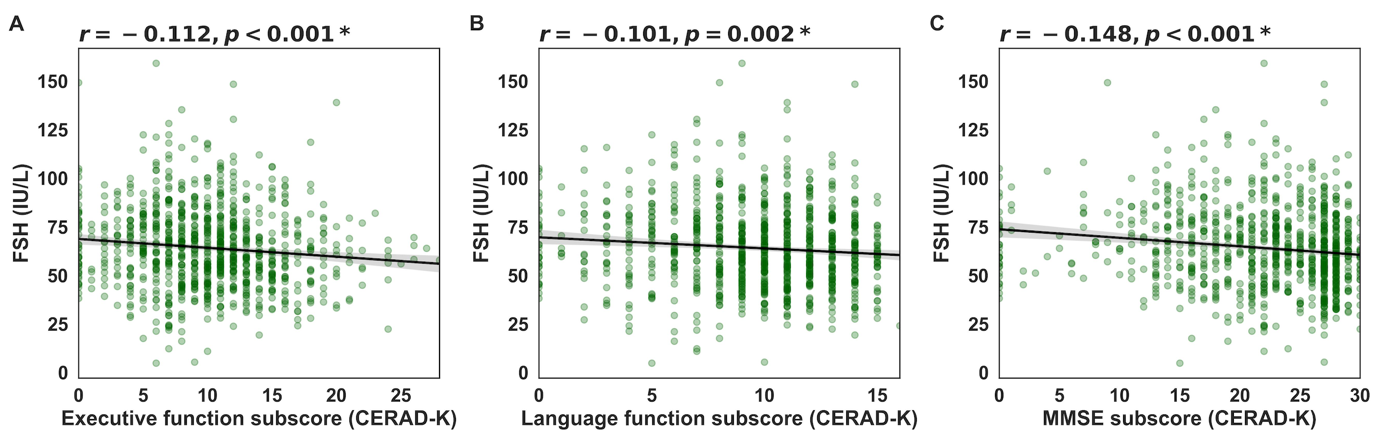
**

**
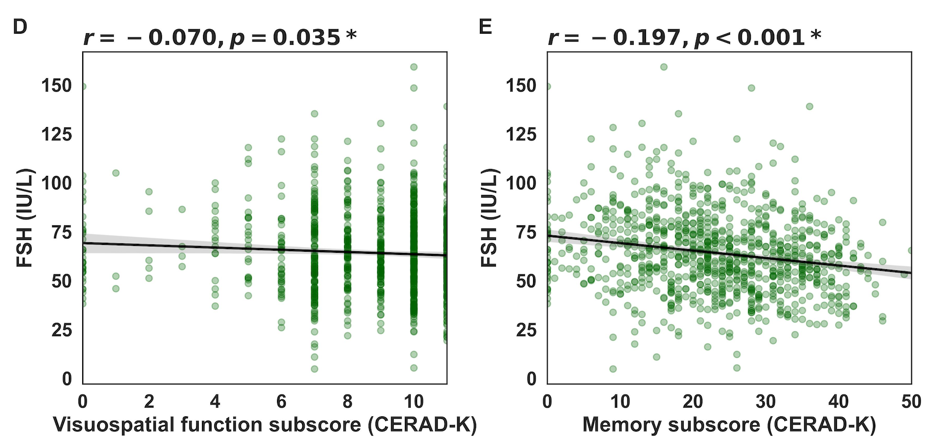
**

^*^ statistical analysis done using partial correlation analysis with age as the covariate

*Abbreviations.* CERAD = Korean version of the Consortium to Establish a Registry for Alzheimer's Disease; FSH: Follicle-stimulating hormone

**Figure S2. Association between E2 with cognitive function and cerebral Aβ deposition**

FSH level did not have statically significant association with A) cognitive function (CERAD-K total scores) and B) global cerebral Aβ deposition**.**

**
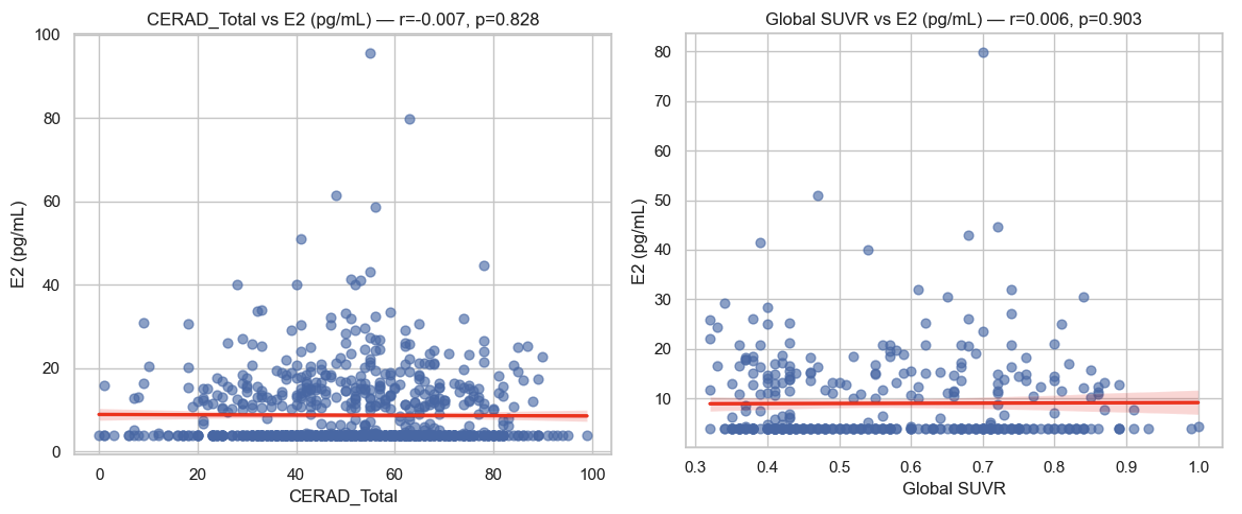
**

*Abbreviations.*; SUVR, Standardized uptake value ratio;

**Figure S3. Association between FSH with cerebral Aβ deposition**

Regional cerebral Aβ deposition or regional SUVR of the A) frontal lobe, B) parietal lobe, C) posterior cingulate cortex/precuneus, D) lateral temporal lobe, and E) anterior cingulate cortex showed a positive correlation with the FSH level.

**
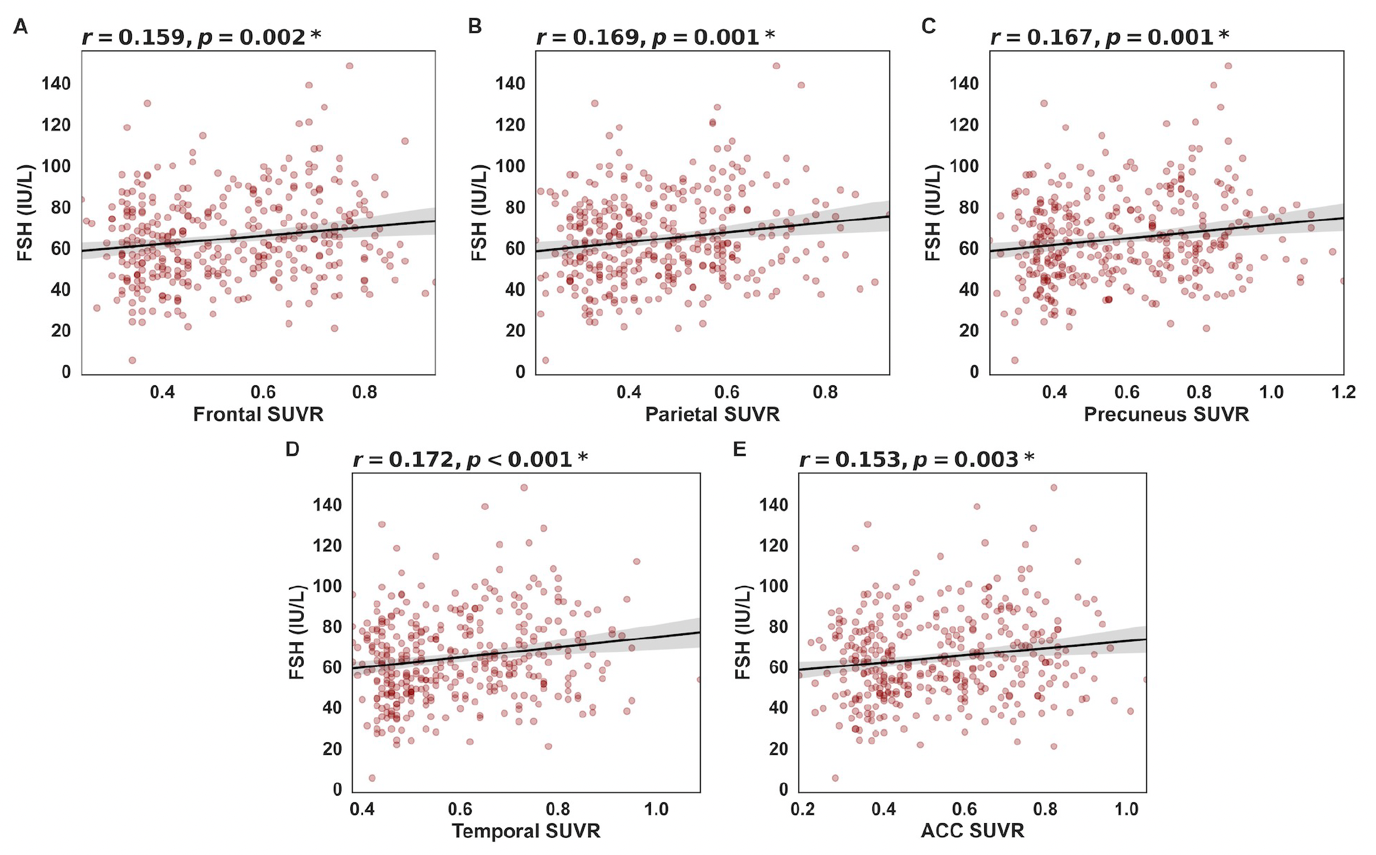
**

^*^ statistical analysis done using Pearson correlation

*Abbreviations.* ACC, Anterior cingulate cortex; FSH, Follicle-stimulating hormone; SUVR, Standardized uptake value ratio;
